# Supplementary material for: Are carbon emissions trading and green financial instruments synergistic? -Comprehensive quantitative research based on content analysis
Source: PLoS One. 2024 Mar 7;19(3):e0298601. doi: 10.1371/journal.pone.0298601 (PMC10919601; doi:10.1371/journal.pone.0298601)
Supplement: S1 Data — (ZIP) [file pone.0298601.s001.zip › Data/地级市Z_F工作报告.zip!/║╙─╧/╔╠╟≡╩╨/└┤╫╘╣1⁄2╓┌║┼í╛┬φ┐╦╩2╛▌═°í┐_2019.docx]

各位代表：

现在，我代表市人民政府，向大会报告工作，请予审议，并请各位政协委员和列席人士提出意见。

一、2018年工作回顾

2018年，面对复杂严峻的国内外形势，在省委、省政府和市委的坚强领导下，全市上下以习近平新时代中国特色社会主义思想为指导，坚持稳中求进工作总基调，全面贯彻落实党的十九大精神和习近平总书记调研指导河南时的重要讲话精神，坚定不移打好高质量发展组合拳，较好完成了市五届人大二次会议确定的各项目标任务。

——发展速度和质量显著提升。全市生产总值完成2389亿元，增长8.7%，增速居全省第1位；固定资产投资增长11.2%，居全省第1位；规模以上工业增加值增长8.7%，居全省第1位；全市居民人均可支配收入增速居全省第1位，超GDP增速1.5个百分点；税收收入增长24.8%，居全省第3位；继永城、示范区之后，民权、虞城、睢阳一般公共预算收入突破10亿元；六大高成长性制造业、高新技术产业增加值分别占规模以上工业增加值的65.1%、40.9%；专利申请量增长54%，居全省第1位；第三产业增加值增长10.9%，居全省第2位，成为拉动经济增长的主要动力。主要经济指标增速有8项居全省第1位，7项居全省前三位，三次产业结构比优化为16:41.3:42.7，发展协调性不断增强，产业结构更趋合理。

——脱贫攻坚取得历史性成就。三年整合财政资金80.2亿元，实现61.7万人稳定脱贫、920个贫困村退出贫困序列，贫困发生率由建档立卡之初的11.5%下降到现在的0.93%，1866名第一书记、3635名帮扶队员奋战一线，党群、干群关系更加密切，农村面貌发生深刻变化，为全面实施乡村振兴战略、决胜全面建成小康社会奠定了坚实基础。

——平台建设取得突破性进展。6个产业集聚区晋升为二星级，永城产业集聚区晋升为三星级；服务业“两区”全部晋星，全省10个三星级服务业“两区”商丘占3席。虞城生态环保电镀中心、夏邑印染产业园等一批创新平台成效凸显，新增睢县、宁陵2个国家火炬特色产业基地。我市被定为国家物流枢纽承载城市。商丘保税物流中心交易额突破10亿元，商丘海关正式挂牌，开启了我市外向型经济发展的新局面。

——服务发展能力显著增强。深入推进“放管服”改革，商丘政务服务网、便民网开通运行，群众借网办事更加方便。在全省率先推出优化营商环境“3350”改革举措。出台金融支持经济发展7个评价体系，新增存贷比达79.7%；社会融资规模272亿元，是2017年的4倍，总量居全省第3位。补充耕地13万亩、供地3.7万亩，土地利用效率居全省第1位，为高质量发展提供了有力的要素支撑。

——民生保障更加有力。全市财政民生支出396.4亿元，为历年来最高，办成了一批群众普遍关注的难事实事。城镇新增就业7.4万人，农村劳动力转移就业5.2万人。新增中小学、幼儿园学生学位1.9万个，医院床位1968张，是近年来增幅最大的一年，有效缓解了入学难、看病难问题，人民群众获得感、幸福感显著增强。

一年来，我们主要做了以下工作：

(一)始终坚持发展第一要务，加快构建现代产业体系。先进制造业发展步伐加快。设立总规模4亿元的专项资金，支持288家企业实施“三大改造”，在全省智能制造观摩点评活动中，获得小组第1名。聚焦产业集聚区“二次创业”，制定考核评价办法和企业分类综合评定办法，引导企业高质量发展，加快了新旧动能转换。全市产业集聚区主营业务收入突破3500亿元、税收突破70亿元，永城、梁园、虞城产业集聚区主营业务收入超过500亿元，民权、夏邑产业集聚区超过300亿元，10个百亿级产业集群主营业务收入全部突破100亿元，食品加工、装备制造产业集群突破1000亿元。全国首个制冷产业指数在民权发布，成为行业发展的风向标。国家级高新技术特色产业基地达到5家，居全省第1位；国家级科技企业孵化器5家，居全省第3位；省级可持续发展实验区5家，居全省第1位；高新技术企业111家，居全省第5位，创新发展步伐进一步加快。先进装备制造产业园发展迅速，神火铝箔等重点项目进展顺利，北汽福田新能源车、天津力神动力电池、海马集团新能源车等项目相继签约落地，招商引资和重点项目建设取得重大突破。农业供给侧结构性改革深入推进。实施以“四优四化”为主要内容的“1112”工程，建成高标准农田521.4万亩，粮食总产突破140亿斤，发展优质花生158.6万亩、优质水果83万亩、蔬菜252万亩，新增“三品一标”特色农产品152个、国家农产品地理标志4个，创建国家级农业标准化示范区5个，居全省第1位。商丘职业技术学院参与完成的高产优质小麦新品种项目，获国家科技进步二等奖。肉蛋奶产量稳定增长，非洲猪瘟防控工作扎实有效。金豆子蔬菜晋升为国家级农业产业化龙头企业。新注册农民专业合作社845家、家庭农场2017家。永城、柘城成功创建国家级一二三产融合发展先导区。全国辣椒产业大会、中部贫困地区农产品产销对接会等成功举办，商丘特色农产品影响力持续提升。现代服务业发展态势良好。传化公路港大数据中心、分拨中心、结算中心投入使用。快递业务量完成1.2亿件，业务收入8亿元，均居全省第3位。电信业务量完成269.6亿元，增长190.2%，被评为“宽带中国”优秀城市。借力资本市场加快发展，春来集团在香港上市，155家企业在中原股权交易中心挂牌。农信社改制工作进展顺利。全市旅游总收入35.7亿元，增长19.2%，“游商丘古都城、读华夏文明史”文化旅游品牌进一步叫响。

(二)始终坚持抓重点补短板强弱项，坚决打赢打好三大攻坚战。聚焦聚力脱贫攻坚。围绕“两不愁三保障”扶贫标准，动员全市各方力量尽锐出战，坚持精准方略，下足“绣花”功夫，注重扶贫同扶志、扶智相结合，坚持脱真贫、真脱贫，做到责任、政策、工作三落实，实施贫困村“九大工程”和贫困户“六改工程”，实现贫困村标准化卫生室、文化广场全覆盖，贫困村基础设施和贫困户户容户貌得到根本性改变。687个扶贫车间和928座光伏电站建成投用，920个贫困村全部消除了集体经济空白，提供7万多个公益性岗位。尤为可贵的是，我们在推进脱贫攻坚中形成了许多实践成果和制度成果，涌现了一大批先进典型，更加坚定了打赢打好脱贫攻坚战的信心决心。广大农民的精神面貌有了显著改善，贫困户和贫困人口、广大农民致富奔小康的内生动力更加强劲。持续抓好环境治理。全力做好中央环保督察“回头看”问题整改，全年空气质量优良天数204天，高于全省平均水平13天，居全省第4位；是豫东南PM10平均浓度唯一完成年度目标任务的省辖市。8个国控断面水质全部达标，为近年来最好水平。加强饮用水水源地保护，完成保护区内居民搬迁，长期影响全市饮水安全的隐患得到彻底整治。在全省污染防治攻坚考核中，水污染、土壤污染防治双双获得优秀等次，受到省政府通报表彰。扎实做好重大风险防范化解。规范政府举债行为，完成政府性债务风险防控目标。依法积极稳妥处置非法集资案件，有效化解了部分区域信用危机和部分企业的股权质押、强制平仓退市风险，一批长期困扰发展的问题楼盘得到解决，为高质量发展创造了稳定有序的社会环境。

(三)始终顺应人民对美好生活环境的新期待，推动城乡面貌不断改善提升。规划编制取得新成果。商丘市城乡总体规划、古城保护性规划获得省政府批准，编制了36个专项规划和各县域城乡总体规划，完成了日月湖商务中心区、高铁核心区等重点区域城市设计。规划体系逐步完善，为城乡有序建设、土地集约利用、空间结构优化提供了科学依据。基础设施建设取得新突破。商合杭高铁全面铺轨，商丘东站站房建设加快推进，沱浍河航运工程进展顺利，综合交通枢纽地位持续强化。全市新建农村公路2431公里，其中虞城完成962公里。中心城区实施庄周大道等4条环城路、神火大道南段等8条主干道以及140条次干道建设，路网结构进一步完善。新建改造供水、供气、供热、污水管网260公里，新增供热面积350万平方米；中电国际两台机组建成运营，结束了中心城区没有大型可靠热源的历史；第六污水处理厂二期建成运行。持续推进综合整治，内河水质不断改善。新增绿地面积402万平方米，“开窗见绿、出门进园”正在变为现实。百城提质和“两城”创建取得新进展。以项目建设为抓手，做好“以水润城、以绿荫城、以文化城、以业兴城”四篇文章，全市开工城建项目684个、完成投资486亿元。夏邑以天龙湖生态综合整治为抓手带动国家园林县城整体提升，睢县被评为“2018百佳深呼吸小城十佳示范城市”，柘城通过国家园林城市初验。全市城镇化率提高1.59个百分点，增幅居全省第2位。以国家卫生城和全国文明城市创建为契机，中心城区新建改造公厕100处，改造提升背街小巷301条，城市基础设施日趋完善，公共服务功能不断提升，人居环境更加舒适宜居。

(四)始终把优化营商环境摆在突出位置，全方位推进改革开放。出台了促进非公有制经济发展系列政策，点燃了全社会创新创造的激情。持续深化行政审批制度改革，5456项审批事项全部实现“一窗受理、一网通办”，投资项目审批事项进一步削减，企业和群众办事慢、办事繁问题得到初步解决。整合全市37家服务热线电话，组建12345政府服务热线平台和“企业投诉平台”，实行全天候服务，办结各类事项5万多件，荣获全国“先锋奖”。国有企业改革扎实推进，被省政府评为2018年度国企改革攻坚工作先进省辖市。信用商丘建设成效显著，在全国综合排名由3月份的第214名提升至年底的41名，由全省倒数第二提升至正数第二。大力实施开放带动战略，实际利用省外资金740亿元，居全省第3位。成功举办第七届国际华商节和食品、制冷等展销活动，为企业开拓市场搭建了广阔舞台。

(五)始终坚持以人民为中心的发展思想，尽心尽力为群众解难题办实事。就业和社会保障体系不断健全。扎实推进退役军人、大中专毕业生和返乡农民工等群体就业创业。持续提高城乡医保、低保、特困人员供养标准和补助标准。群众普遍关注的安置房建设成效明显，中心城区竣工4.4万套、520万平方米，近12万群众喜迁新居。在全省率先开展“双百一千”工程，100个社区服务中心、104所敬老院功能得到提升完善，群众办事更方便，老年人生活更舒心；1077个农村儿童之家成为留守儿童的心灵家园。教育事业全面发展。“全面改薄”如期完成，新建改扩建农村寄宿制中小学91所、公办幼儿园58所。商丘工学院、市一高、一中新校区喜迎新生。柘城、宁陵、民权和梁园顺利通过全国义务教育发展基本均衡县认定。职业教育和高等教育水平进一步提升。文化事业繁荣发展。市文化艺术中心主体工程完工，建成村级文化服务中心3427个，组织系列文化惠民活动4万余场，人民群众享受到更多均等的公共文化服务。《文化商丘》丛书编撰完成。商丘古城修复性保护与展示、宋国故城考古发掘、大运河遗址商丘南关段等重点文化遗产保护工作有序开展。健康商丘深入推进。深化“三医”改革，公立医院全部取消药品加成，家庭医生签约服务全面展开，“两癌”“两筛”和慢性病综合防控示范区建设位居全省前列，市第一人民医院心血管分院等医疗项目建成投用。开展食品药品安全综合整治，确保了人民群众饮食用药安全。平安商丘建设持续深化。深入开展扫黑除恶专项斗争，依法打击各类违法犯罪，确保了社会治安大局持续平稳。畅通信访渠道，坚持领导干部定期接访，化解了一批信访积案。深刻汲取“12·17”火灾事故教训，全面开展安全生产事故隐患大暗访大排查大整治大执法攻坚行动，安全生产风险防控能力得到新提升。面对台风“温比亚”带来的百年不遇特大暴雨，全市上下迅速行动，有效处置各类灾情险情，确保了人民群众生命财产安全，以实际行动谱写了一曲众志成城、奋勇抗灾的时代凯歌。

(六)始终把政府自身建设摆在突出位置，汇聚加快发展强大力量。树牢“四个意识”，坚定“四个自信”，做到“两个维护”，始终同以习近平同志为核心的党中央保持高度一致。主动接受市人大及其常委会的法律监督和市政协的民主监督，认真听取各方面的意见建议，办结人大代表建议和政协委员提案745件。始终把纪律和规矩挺在前面，全面抓好中央和省委巡视反馈意见整改，严格落实中央“八项规定”精神及实施细则，坚决纠正“四风”，持续改进作风，政府治理能力不断提升。

积极培育和践行社会主义核心价值观，进一步传播“商丘好人”精神，电影《李学生》在全国院线公映，弘扬了社会正能量。破除陈规陋习、崇尚文明新风，倡导一切从简的婚丧礼俗，得到民政部好评。持续加强国防教育、国防后备力量及人民防空建设，驻商部队为地方发展作出重要贡献，军民融合深度发展。积极支持工会、共青团、妇联等人民团体发挥作用。第四次经济普查、“七五”普法深入推进。对台、史志、供销、盐业、档案、审计、残疾人康复、民族宗教和对口支援等工作都取得了新成绩。

各位代表！这些成绩的取得，是省委、省政府和市委坚强领导的结果，是市人大、市政协有效监督、大力支持的结果，是全市上下撸起袖子干出来的结果，是新时代广大奋斗者挥洒汗水拼出来的结果。在此，我代表市人民政府，向全市人民，向驻商解放军、武警官兵，向各民主党派、工商联、人民团体和无党派人士，向各位人大代表、政协委员，向所有关心支持商丘发展、参与商丘建设的朋友们，致以崇高的敬意和衷心的感谢！

各位代表！回顾一年来的奋斗历程，我们深深体会到：只有始终坚持把党的政治建设作为统领，以党的建设高质量推动经济发展高质量，才能够进一步激励广大干部新时代新担当新作为。只有始终坚持发展是第一要务，致力于加快发展速度、提升发展质量、补齐发展短板，才能够一步步把商丘的宏伟蓝图变成美好现实。只有始终坚持高质量发展根本要求，增强发展的整体性，提高发展的协调性，才能够有效解决商丘发展不充分不平衡的主要矛盾。只有始终坚持把防风险放在更加突出位置，牢牢守住风险防控底线，才能够为发展创造良好的环境。只有始终坚持以人民为中心的发展思想，坚持办好事关人民群众切身利益的实事好事，才能够真正得到人民群众的拥护和广泛参与。只有始终坚持发扬艰苦奋斗作风，才能够在全市上下形成思发展谋发展的浓厚氛围，汇聚成推动发展、干事创业的强大合力。

在肯定成绩的同时，我们也清醒地认识到，我市经济社会发展还存在一些困难和问题：一是跨越发展任务艰巨。人均生产总值和财力相对较低，城乡基础设施、公共服务设施、环境基础设施相对薄弱，缩小与全国全省平均水平差距压力较大，民生领域还有不少短板。二是转型发展任务繁重。科技创新能力较弱，科技投入和人才不足，战略性新兴产业和高新技术产业规模不大，带动能力不强。三是实体经济发展面临困难。融资难、融资贵问题依然突出，用工、用能等要素保障趋紧，民营经济活力不强，外向型经济发展不足。四是营商环境需进一步改善。简政放权没有完全到位，一些部门和干部主动服务的意识不强，干事创业的热情不高，担当作为的勇气不足。对此，我们将坚持问题导向，采取有效措施加以解决，努力把各项工作做得更好。

二、2019年工作总体要求和重点任务

2019年是新中国成立70周年，是全面建成小康社会、实现第一个百年奋斗目标的关键之年，也是商丘实现所有贫困县摘帽、以新姿态站在新起点，奋力推动高质量发展、全面实施乡村振兴战略至关重要的一年，做好今年工作意义重大。

当前，全球经济形势更加严峻复杂，国内经济下行压力加大。但是，我国经济长期向好的态势没有变，经济发展由出口导向型向内需拉动型转变，对外开放的大门越开越大，创新引领发展的步伐越来越坚实，高质量发展的路子越走越宽。我们必须把握新趋势，理解新内涵，充分认识到商丘正处在转型发展的关键时期。我们必须紧盯建设国家区域中心城市的目标，认清新形势、适应新转变、顺应新要求，从宏观大势中掌握主动，从政策利好中抢抓机遇，从自身条件中发挥优势，把握新规律，壮大新动能，抢占制高点，实现新突破，持续保持跨越发展的态势，全力推动经济社会高质量发展。

2019年政府工作的总体要求是：以习近平新时代中国特色社会主义思想为指导，全面贯彻党的十九大和十九届二中、三中全会精神，深入落实习近平总书记视察河南时重要讲话和在全国“两会”上河南代表团重要讲话精神，按照中央经济工作会议、省委省政府和市委决策部署，统筹推进“五位一体”总体布局，协调推进“四个全面”战略布局，坚持稳中求进工作总基调，坚持高质量发展根本要求，以供给侧结构性改革为主线，以新发展理念为引领，持续推进“四个着力”、打好“四张牌”，围绕“四个总”总体要求和“4233”奋斗目标，统筹推进稳增长、调结构、促改革、惠民生、防风险、保稳定工作，着力落实“六稳”要求和“八字”方针，把实施乡村振兴战略、做好“三农”工作放在经济社会发展大局中统筹谋划和推进，扛稳粮食安全这个重任，持续打赢打好三大攻坚战，强力实施以党的建设高质量推动经济发展高质量“一方案十专案”，努力保持经济高质量发展和社会大局稳定，在中原更加出彩征程中迈出更大步伐，为全面建成小康社会收官打下决定性基础，以优异成绩庆祝中华人民共和国成立70周年。

2019年我市经济社会发展的主要预期目标是：继续保持“三个高于”“三个同步”，生产总值增长8%左右；全员劳动生产率增长6%左右；全社会固定资产投资增长10%以上；一般公共预算收入增长8%以上；社会消费品零售总额增长10%以上；外贸保持平稳增长；居民人均可支配收入增长8%；居民消费价格指数涨幅控制在3%以内；常住人口城镇化率提高1.5个百分点；力争研究与试验发展经费投入强度1%以上；城镇调查失业率、登记失业率分别控制在5.5%、4%以内；单位生产总值能耗下降3%以上，节能减排等约束性指标完成省下达任务。

落实总体要求，实现预期目标，我们将按照市委五届八次全会确定的工作任务，着力抓好十个方面的重点工作：

(一)着力优化营商环境，激发市场主体活力。营商环境是高质量发展的生命线。今年，我们将以减税降费政策落地、金融支持实体政策进企、大幅压缩行政审批时限为重点，进一步提高办事效率、提升服务能力。持续深化“放管服”改革。大力推进行政许可权和管理事权下放，重点向示范区下放经济发展审批权，向两个区下放城市管理权，向省级经济发达镇下放县级经济管理权，通过“放”激活干事创业的动力，激发创业创新的热情。全面实施“双随机、一公开”监管模式，通过“管”营造公平透明、竞争有序的市场环境。以“刀刃向内”的勇气，重塑审批流程，压缩审批时限，在工业类工程项目50个工作日完成审批的基础上，所有建筑类工程项目100个工作日内取得施工许可证，所有工程审查只交“一套图纸”，所有竣工验收一次性完成，以政府审批时间的压减实现企业投资成本的降低。深入推进“互联网+政务服务”，坚决治理“一窗受理、一网通办”中的“名进实不进、人进权不进”现象，决不让服务窗口变成“收发室”。依托12345政府服务热线，设立“企业服务110”，每年开展1—2次“企业评议部门”活动，排名结果公开，连续靠后的问责，通过“服”让各类市场主体敢于创业、放心投资、踏实经营。持续增强实体经济发展活力。坚持“两个毫不动摇”，对减税降费政策坚决做到应减尽减、应降尽降、应享尽享。对企业不动产转让、股权资产过户等事项，建立绿色通道，特事特办，支持企业盘活资产。对困难企业制定“一企一策”帮扶措施，增强解困的针对性和有效性。持续加大金融服务实体经济力度。完善金融机构7个考核评价体系，鼓励银行对更多符合条件的企业实行无还本续贷和永续贷。以新增贷款主要用于实体经济的具体行动，支持管理规范、经营有方、成长性强的企业加快发展。加强金融监管，清理中介服务收费，落实利率只减不增、贷款无额外附加、放款足额及时等政策措施，在解决融资贵问题上让企业有实实在在的获得感。今年市财政将列支不少于1亿元，各县(市、区)不少于3000万元，健全企业增信和风险补偿机制，真正使企业感受到政策的红利、环境的便利、服务的给力。持续加强信用体系建设。整合税务、市场、公安、法院、社保等企业信用信息，健全企业信用综合信息平台，完善查询系统，增进银企沟通，使企业融资更便捷、更高效。继续强化守信联合激励、失信联合惩戒工作机制，加强对县(市、区)不良贷款率的考核评价，严厉打击恶意逃废银行债务行为，创造良好的金融生态环境。开展政府招商引资各项承诺清理行动，决不能“新官不理旧账”，以政府诚信带动个人诚信和社会诚信。

(二)巩固提升脱贫攻坚成效，强力实施乡村振兴战略。按照“五个振兴”的总要求，谱写乡村发展的新篇章。巩固提升脱贫攻坚质量。认真开展脱贫攻坚工作“回头看”，有效防止返贫和出现新的贫困。以产业扶贫为重点，建立农民稳定增收的长效机制。切实加大特殊贫困群众扶持力度，通过多种渠道为贫困群体提供兜底保障，以乡村全面振兴带动脱贫质量全面提升，确保贫困人口在小康路上一个都不掉队。坚决扛稳粮食安全这个重任。持续推进高标准农田建设，稳定粮食种植面积，加快对商麦系列的研发投入和成果应用，把“藏粮于地、藏粮于技”落到实处，不断提高粮食的产量和质量，确保粮食综合生产先进位次。延伸粮食产业链、提升价值链、打造供应链，不断提高种粮收入，激发种粮积极性，以新担当新作为持续提升粮食安全保障能力。按照“四优四化”标准，以品牌建设为重点，继续实施农业供给侧结构性改革“1112工程”，加快“三品一标”农产品认证，把商丘特色农产品做优做强。坚持“农头工尾、粮头食尾”，提高农业产业化水平，培育壮大农业产业化龙头企业，扶持发展新型农业经营主体，促进农户分散经营与现代农业有机衔接，提升农业生产组织化、专业化程度和生产经营效益，奋力推动由“豫东粮仓”到“国人厨房”再到“世人餐桌”迈进。编制好乡村振兴战略规划。按照编制多规合一实用性规划的要求，以产业发展为根本，以生态宜居为核心，以历史文化传承为基础，以集约有序为关键，编制乡村振兴规划体系，明确“四类村”定位，合理安排各类建设用地空间布局，彰显特色浓郁的豫东乡村风貌。各县(市、区)6月底前完成村庄规划编制，为乡村振兴战略有序实施提供战略遵循。扎实推进农村人居环境综合整治。借鉴浙江“千万工程”经验，健全农村环卫长效机制，统筹建设污水、垃圾、粪便处理系统，解决“厕所革命”后续问题。深入推进“四美乡村”建设，以“三清一改”为主要内容，高标准抓好示范县、示范乡镇、示范村建设，确保高质量打赢打好乡村振兴首场战役。补齐乡村振兴短板。以“四好农村路”建设为载体，加快完善县域乡村公路网络，健全乡村客货运场站服务体系。统筹抓好农田水利、安全饮水、乡村物流、宽带网络等基础设施建设，逐步建立全域覆盖、普惠共享、城乡一体的基础设施服务网络。制定长远计划，确定年度目标，扎实开展农村土地综合整治，破解土地、资金难题。加强土地执法监管，坚决依法整治农村“双违”，牢牢守住耕地保护红线。始终把人作为乡村振兴的关键要素，营造返乡创业的优良环境和条件，吸引外出经商、务工人员回乡就业创业，加快培养一批懂农业、爱农村的工作队伍和懂技术、善经营的新型职业农民，打造乡村振兴的生力军。

(三)深入实施创新驱动战略，推动制造业高质量发展。制造业是经济发展的根基。坚持以高端化、绿色化、智能化、融合化为目标，实施智能制造引领、先进技术应用与创新、传统产业提质、新兴产业培育四大行动，加快先进制造业2025建设步伐。加快产业集聚区建设。按照质量第一、效益优先的原则，促进产业集聚区“二次创业”。加快印染产业园、生态环保电镀中心二期、食品生态环保中心、力量钻石研发中心等产业集聚区创新平台建设，推动产业集聚区向更大规模、更高层次发展。引导企业和高校把实验室、研发中心、加工中心作为公共平台对其他企业开放，实现由错位发展向错位与协同发展并行转变，在产业链、创新链和价值链上加快形成节约共享、互补协同的发展局面。继续推进“三大改造”。突出抓好“五个五”重大工业项目，深入推进智能化改造提效扩面、绿色化改造提标示范、技术改造提速升级。把今年作为“三大改造提升年”，再完成269家规模以上工业企业技术改造任务，使改造升级的新型企业占规模以上工业企业的40%以上。支持金振源电子、足力健鞋业、嘉鸿鞋业、永安纺织等智能车间和生产线示范化建设，推动电子信息、制鞋产业向数字化、智能化方向发展；加快华晶金刚石二期、宝晶新材料等项目建设，推动金刚石制品向高端化、多样化发展；加快阿诗丹顿、冰熊冷藏车、康拜恩电器等扩产能项目建设，推动家用电器等行业向产品绿色化转型升级。增强制造业技术创新能力。聚焦培育“四个一批”，实施创新龙头企业提升引领、高新技术企业倍增、科技小巨人培育、创新驱动提速增效工程，积极对接国内外智能制造先进技术和智力资源开放创新平台，争取引进一批智能产业重大项目。加快推进纺织新材料研究院、钻石院士工作站、制冷博士后科研流动站、新能源汽车研究院建设，加强先进技术创新攻关，申报高新技术企业30家、省级工程技术研究中心15家。深入开展“质量标杆进企业活动”，支持企业增品种、提品质、创品牌。大力弘扬劳模精神和工匠精神，建设一支知识型、技能型、创新型劳动者大军，为经济发展提供持续动力。抓好新兴产业培育。确保北汽福田新能源车年底前具备试生产条件，神火双零铝箔、恒天永安一期50万锭智能高端化纤纺、年产20万吨乙二醇项目、美兰生物制药、三爱司光电耦合器、爱德时代新能源柔性电池等重大新兴产业项目年内竣工投产。积极推进恒天重工无纺布生产线、海马新能源汽车、天津力神动力能源等项目开工建设。完善数字基础设施，建设商丘大数据云中心，推动大数据产业发展，为高质量发展助力添彩。

(四)加快现代服务业发展，增强引领服务经济发展的动力和活力。全力推进国家物流枢纽承载城市和全国供应链创新与应用试点城市建设，努力开拓内需市场，聚集人气与商气，不断满足人们对美好生活的消费需求。打造现代服务业发展高地。聚焦日月湖现代服务业集聚区，加快推进建业总部港、金融创新大厦、城乡规划设计中心、绿地星级酒店群等项目建设进度，加快发展楼宇经济，吸引总部经济、中介服务、现代金融等高端服务业入驻，提升产业层级，适应高质量发展要求。聚焦高铁商务区，加快推进爱琴海购物公园、绿轴休闲步行街和高铁广场D区等项目建设进度，增强高铁核心区的带动能力。聚焦古城历史文化创意创新区，坚持基础建设与业态培育并重，加快推进商丘古城修复性保护与展示工程，启动5A级景区创建，争取更多项目建成开放。以服务业“两区”为载体，加快县域城市综合体建设，培育科技服务、现代物流、电子商务等业态，形成县域现代服务业集聚核心。加快培育现代化商品交易市场。力争利用3年左右时间，做好商丘农产品中心批发市场和建材、家电等大宗商品专业市场外迁及转型升级工作。按照未来业态发展模式和交易方式，重点培育4—6个有规模、有影响力、有带动力的市场集聚区，构建有商丘特色的商品交易市场体系。立足农业发展实际，优化农村市场布局，大力发展辣椒、果蔬、种子、农资等专业化、品牌化农产品交易市场，促进农业和农村繁荣发展。大力发展电商和现代物流业。持续把电商和物流业作为枢纽经济的先导，以冷链物流、快递物流和电商物流为重点，加快推进传化公路港智能化仓储、华东商储等项目建设，积极引进拥有全国全球物流网络的知名企业。对接京东、阿里巴巴、拼多多等电商平台，积极引入顶级供应链企业，抢占电子商务发展制高点，拓展优势产品的营销范围。加快惠龙易通智慧物流园项目建设，带动流通运输业提升规范化、信息化、标准化水平。发挥电子商务进农村示范县的优势，大力发展农村电商，使千家万户农民借助互联网成为特色农产品的经营者。加快发展各具特色的文化旅游业。大力发展历史文化游，深入挖掘历史文化遗址遗迹，把历史遗存作为旅游资源，谋划精品线路，打造特色鲜明的文化旅游体系。大力发展民间传统文化游，培育“民权虎”、火店宫灯、刘腾龙毛笔、木兰剪纸等特色文化旅游产品，让民间传统文化活起来，把老祖宗留下的“宝贝”用起来，推动优秀传统文化创造性转化、创新性发展。大力发展特色农产品休闲游，以黄河故道生态走廊为重点，结合草莓、酥梨、葡萄等特色农产品，发展乡村采摘体验、生态休闲旅游。大力发展创意文化游，加快华强方特项目建设，带动以动漫为主题的创意文化旅游业发展，打造文化体验与旅游消费的新载体、发展经济的新热点。大力发展红色革命教育游，以淮海战役为主线，以陈官庄淮海战役纪念馆、淮海战役总前委旧址等为重点，叫响商丘红色旅游品牌。

(五)以百城建设提质工程为载体，扎实推进新型城镇化进程。强化产业支撑，完善城市功能，推动生产、生活、生态融合发展。调整优化城市功能布局。落实好商丘市城乡总体规划，实施“东拓、西延、北优、南控”，形成“三心辉映、多轴联动、绿廊楔入、片区融合”的城市空间布局结构，以组团式发展布局破解城市蔓延难题，建设宜居宜业、富有特色、充满活力的现代城市。以交通一体化为先导，推进形成商虞、商宁半小时生活圈，加快“一中心两组团”融合协同发展步伐，打造商丘发展核心增长极。提升中心城区综合承载力。坚持“窄马路、密路网”的原则，实施归德北路和迎宾东路等127条道路、运河南京路桥等39座桥梁、5座闸坝工程项目。加快水气暖等基础设施建设，完成四水厂20万吨扩建工程，碧源水厂10万吨新建工程主体完工。完成供热环形主管网建设，新增供热面积500万平方米。确保二污6月底前建成运营，改造积水点48处，有效解决城区内涝问题。按照“五湖七廊九带”的布局，加快推进日月河公园和9个带状公园一期工程、3个湿地公园、12个便民公园、30个游园绿地建设，完成人民公园升级改造，新增绿化面积1066万平方米。推进城市精细化管理。以城市“四治”为目标，持续推进环境治理的信息化、法治化、标准化建设，健全城市管理科学化、精细化、智能化的法治环境和制度体系。以“两城”创建为载体，开展交通秩序治理、背街小巷改造、生态绿化提升、城市扬尘治理专项行动，让蓝天常在、绿水常流，让城市更加整洁有序。增强县城带动能力。以百城建设提质工程为抓手，全年实施百城提质项目659个，完成投资613亿元，重点做好宁陵老城区有机更新、虞城棚户区改造等重点项目建设，提升优化老城区的功能与环境，使新老城区更加均衡发展。突出抓好县城教育、医疗、生态等重大公共项目建设，制定更加灵活的政策，促进城乡要素高效流动，努力把县城打造为承载产业和吸纳就业的重要载体。永城要加快提质提速转型发展，发挥好副中心城市的作用，持续当好商丘县域发展的排头兵。统筹兼顾建设特色村镇。以重点镇建设为引领，突出发展足力健幸福小镇、石桥梨花小镇、牛城辣椒小镇等24个工业强镇、商贸重镇、旅游名镇，努力把镇区打造为市政基础设施和公共服务设施向农村延伸的载体。加快历史文化和自然风貌特色村的认定，着力中心村功能建设，发展以中心村为支点、自然村分布合理、产业优势突出、风貌特色鲜明的美丽乡村。

(六)加快推进重大基础设施建设，增创经济发展新优势。牢牢抓住中央实施积极的财政政策机遇，加快交通、能源、通信等重大基础设施建设，解决资金瓶颈，补齐发展短板，拓展发展空间。加快编制综合交通枢纽规划。科学规划以商丘为中心，辐射周边的铁路、公路、航空、河运交通网络，合理布局各种运输方式“无缝衔接、零换乘”的多式联运体系，强化枢纽能力、配套能力、承接能力、场站能力建设，构建对外开放和物流大通道，形成“一网六中心”枢纽格局。加快综合交通枢纽设施建设。积极配合商合杭高铁建设，确保年底前试运行；积极推动京雄商高铁和三洋铁路早日动工建设；按期完成高铁动车所、商丘站南站房、商丘东站站房各项建设任务；积极谋划商丘到新郑机场、商丘到周口等城际铁路规划建设的前期工作；力争早日开工商丘机场改扩建工程，全面启动沱浍河航运二期工程和港口设计工作。加快推进3个环城高速出入口、阳新高速和“三环九放射”一期工程建设，形成支撑枢纽地位的骨干工程体系。加快信息基础设施建设。高度重视人工智能、工业互联网、物联网、5G通信网络等新型基础设施建设，完成总投资10.8亿元的无线网络和专网基站等建设任务，确保2020年实现5G连续覆盖、具备商用条件的目标。依托智慧商丘，积极推进智慧政务、智慧交通、智慧城管、智慧环保等建设，运用信息技术等科技手段，切实提高城市管理、运营和服务水平，让人民群众放心、安心、舒心。

(七)全面提升生态文明建设水平，让绿色成为发展主色调。牢固树立绿色发展理念，坚持削减污染排量与扩大环境容量“两手抓”，生态保护、建设、治理“三管齐下”，认真实施“四大行动”，以严格责任追究倒逼工作落实，推动生态文明建设再上新台阶。坚决打赢蓝天保卫战。坚持优化“四个结构”，加快重污染企业退城入园，推进商砼搅拌站外迁，持续抓好散乱污企业专项治理、重污染天气管控等一系列行之有效的措施，确保全市PM10、PM2.5平均浓度和空气优良天数完成省定目标，使“商丘蓝”更加清澈宜人。全面打好碧水保卫战。加快实施“四水同治”，认真落实河(湖)长制，实施流域面积100平方公里以上42条河流整治三年行动计划，确保出境水质稳定达标。巩固水源地综合整治成果，让老百姓喝上更加优质的放心水。深入开展净土保卫战。加强自然资源保护和开发，促进各类资源高效利用。推行秸秆还田、粮经轮作等措施，保持化肥、农药使用“零增长”，确保土壤环境质量。持续推进国土绿化提速行动。按照“一带一网两环五区多廊道”布局和“六化”要求，提升平原绿化和森林商丘建设水平，提高绿化标准和生态效果。完成造林9.7万亩、森林抚育10.3万亩，新创2—3家省级以上森林公园，构建以黄河故道森林公园为主体、湿地公园相结合、绿脉相连的森林生态体系，让豫东大地绿起来、美起来。

(八)坚持改革不停顿开放不止步，持续增强经济发展动力活力。积极融入“一带一路”和“三区一群”战略，推动形成全面改革开放新格局。推进重点领域改革。深化财税体制改革，完善规范透明、标准科学、约束有力的预算制度，实现预算和绩效管理一体化。牢固树立过紧日子的思想，优化财政支出结构，一般性支出压减5%以上，“三公经费”支出再压减3%左右，节余资金全部用于民生保障；对进度慢的项目资金、部门结余和结转两年以上的资金，一律清理收回统筹使用。以“三块地”改革为抓手，统筹推进农村综合改革，为乡村振兴增添动力。做好行政执法体制等改革事项。创新投融资体制机制，发挥财政资金杠杆作用，支持市发投、市政投、市交投等市场化改革，完善立、融、建、管、还的项目建设机制。积极融入区域发展大局。按照国家区域中心城市定位，强化商丘在全省经济布局中的功能作用，顺应区域竞合趋势和规律，从重要战略机遇期内涵变化和区域经济合作中把握新机遇，在新一轮的区域竞争中争取主动、抢占先机。向东主动对接长三角，向西积极融入“三区一群”战略，向南紧密跟进粤港澳大湾区，向北密切联系京津冀和雄安新区，加强产业合作，形成连贯东西、沟通南北的发展大局。持续加大招商引资力度。依托产业集聚区、服务业“两区”等载体，引进产业运营商，打造全方位产业服务平台，全面提升整体竞争力和项目吸引力。创新招商模式，注重发挥政府基金、产业引导资金等撬动作用，开展以商招商、产业链招商，提高招商引资的精准度，每个县(市、区)至少要落地2个5亿元以上、3个3亿元以上产业项目，为产业转型升级、推动高质量发展注入强大动能。推动高水平开放。充分发挥商丘海关业务功能扩展的优势，加快口岸查验区项目建设，争取商丘保税物流中心业务增长50%以上。按照省部协议，依托商丘北站加快铁路物流基地和一类口岸建设，积极支持民权保税物流中心建设，争取综合保税区申报取得实质性进展，如期开展跨境电商业务，尽快形成区域性跨境电商交易“东大门e贸易”中心。加强与郑州航空港、青岛港、连云港等港口合作，积极与郑州国际陆港集团衔接，争取开通中欧班列“商丘号”，更深层次融入“一带一路”，力争国家物流枢纽承载城市建设走在全国前列，推动外向型经济发展得更好，使商丘的优质农产品、特色产品走向世界。

(九)大力发展社会事业，提高公共服务水平。以民生项目建设为抓手，确保民生投入只增不减，坚持办好事关人民群众切身利益的民生实事。办好人民满意的教育。深入实施第三期学前教育行动计划，完善市县乡村四级办园网络，促进学前教育规模与内涵同步发展。进一步优化城乡中小学校布局，中心城区新建中小学25所，开工建设市回中、市一实小新校区，改扩建农村寄宿制学校60所，落实好进城务工人员随迁子女入学政策，使更多孩子享受到更加公平更有质量的教育。大力发展职业教育，促进校企深度合作。持续推进商丘医学高等专科学校升本工作。积极引进高等院校和科研院所。强化综合治理，维护校园安全。加强教师队伍建设，改善教师工作生活条件，让教师成为最受尊崇的职业。推进公共文化服务体系建设。继续开展“舞台艺术送农民”“百戏进百乡”和农村公益电影放映等活动，丰富人民群众文化生活。确保市文化艺术中心如期建成投用，“三馆一中心”项目开工建设。加强文物保护利用，强化文化遗产发掘、保护和传承。加快健康商丘建设。深化“三医”联动改革，做好传染病、慢性病等重大疾病防控工作，抓好中医药传承创新。加快推进市第一人民医院肿瘤治疗中心、市第一妇幼保健院、利君医院等5个医疗项目建设，引进2个医疗院士工作站，加强乡村医生队伍建设，不断破解群众看病难、看病贵问题。结合市中医院新院区建设，规划建设集康复、养老、保健等于一体的健康产业园。推进健康养老产业转型发展，鼓励社会资本投资康养产业，唱响“长寿之乡”品牌，利用各种适宜康养资源，形成特色化、多层次、适应市场需求的康养体系。强化食品药品市场监管，积极推动食品安全示范市、县创建。广泛开展全民健身运动，夯实群众健康基础，形成强身健体、愉悦身心、激发活力的良好氛围。

(十)切实保障改善民生，增强人民群众幸福感、获得感、安全感。民之所盼，政之所向。我们将把工作出发点和落脚点放在保障改善民生上，让发展成果更多更公平惠及人民群众。扎实做好就业和社会保障。坚持就业优先，促进高校毕业生、退役军人、农民工等重点群体就业，建立城乡低保动态调整机制，全面实施80岁以上老年人高龄补贴，巩固扩大各项社保覆盖面。落实“三个聚焦”，做好特困人员供养和临时救助，实施新一轮“双百一千”工程。加快12个便民服务中心建设，构建5分钟、10分钟便民生活圈。加快安置房建设。按照科学有序的原则，继续推进棚户区改造，坚持住房与配套工程同步推进，加快安置小区建设进度，基本建成5.5万套646万平方米，确保8.1万名群众回迁安置，力争更多被征迁群众早日迁入新居。坚持稳地价、稳房价、稳预期的目标，完善住房市场体系和住房保障体系，加大问题楼盘的解困力度，促进房地产业平稳健康发展。加强和创新社会治理。学习推广新时代“枫桥经验”，提升基层社会治理能力。强化源头治理，依法解决群众信访诉求。持续抓好“七五”普法、法律援助等工作。加强社会治安综合治理，深入推进扫黑除恶专项斗争，依法打击传销、非法集资和电信网络诈骗，保持对违法犯罪的高压态势，坚决守护好人民群众的平安生活。健全应急管理体制机制，落实安全生产责任制，加快推行双重预防体系建设，增强重大突发事件处置和防灾减灾救灾能力，持续开展“四大行动”和“霹雳行动”，确保安全生产形势总体平稳，为人民群众创造安全、祥和的生活环境。

大力践行社会主义核心价值观，深入开展文明城市、文明单位和群众性精神文明创建活动，倡导文明乡风、良好家风、淳朴民风，提升全民文明素养。加强国防教育、国防动员、国防后备力量建设和人民防空工作，加快推进退役军人服务保障体系建设，巩固军政军民团结，争创全国双拥模范城“八连冠”。全面贯彻党的民族政策和宗教工作基本方针，促进民族团结、宗教和睦。加强统计工作，做好第四次全国经济普查。积极支持工会、共青团、妇女儿童等群团组织工作，统筹推进外事、侨务、对台、气象、供销、档案、公共资源交易和对口支援等各项工作。

各位代表！“求木之长者，必固其根本。”基础能力建设是一个地方核心竞争力的构成要素。我们将着力抓好事关商丘长远发展的四大战略性工程：大力实施引才入商工程。人才是推动高质量发展的第一资源。人才已成为制约商丘高质量发展的重要因素。我们将坚持党管人才的原则，出台引进人才的各项优惠政策，创造更多让人才施展才华的机会和舞台，形成让人才舒心生活、愉快工作、智慧迸发的环境和生态，以情感为纽带建立“归巢”机制，引导各类人才在商丘就业创业，让更多的人才在商丘大显身手、放手拼搏。大力实施引水入商工程。水源建设是惠及900多万商丘人民的重大民生工程，是支撑城乡可持续发展的战略资源。我们将按照“节水优先、空间均衡、系统治理、两手发力”的原则，谋划实施“南水北调”入商工程，启动引江济淮和引黄入商工程，建设亿立方米级储蓄调节水库工程，形成用水“三线三水源”保障格局。大力实施引电入商工程。电力建设是经济高质量发展的先锋，是提高人民生活质量的基础性资源。我们将积极实施“青电入商”“疆电入商”工程，谋划一批500千伏、220千伏骨干输变电站和高压输电网络建设，力争早日把商丘建成坚强智能电网和泛在电力物联网城市。大力实施引金入商工程。金融是现代经济的血脉，金融强则经济强，金融活则经济活。我们将不断创造适宜金融业聚集发展的环境和条件，积极引进金融豫军以及浙商、广发、光大等金融机构，加大保险、证券特别是天使、风投、股权类基金的招引力度，提高商丘现代金融的聚集度，为企业发展壮大营造优良的金融环境。突出抓好这四大战略性工程，增强经济社会可持续发展的基本要素保障，构建起商丘未来发展的战略支撑，推动商丘在高质量发展道路上行稳致远。

三、全面加强政府自身建设

各位代表！新时代赋予新使命，新时代呼唤新担当，新时代需要新作为。我们将始终坚持党的领导，坚持以人民为中心，加强自身建设，强化责任担当，努力建设人民满意的政府。

旗帜鲜明讲政治。“讲政治是马克思主义政党的根本要求。”我们将高举习近平新时代中国特色社会主义思想伟大旗帜，牢固树立“四个意识”，坚定“四个自信”，做到“两个维护”。切实加强全市政府系统党的政治建设，筑牢信仰之基、补足精神之钙、把稳思想之舵，以实际行动确保党中央国务院、省委省政府和市委各项决策部署落到实处，确保各项工作沿着正确方向不断前进。

依法履职尽责任。“奉法者强则国强，奉法者弱则国弱。”我们将忠诚履行宪法和法律赋予的职责，强力推进法治政府建设，严格按照法定权限和法定程序行使权力，依法接受人大及其常委会的监督，自觉接受政协民主监督，主动接受社会和舆论监督，让权力在阳光下运行。坚持依法科学民主决策，提高运用法治思维和法治方式深化改革、推动发展、维护稳定能力，努力形成办事依法、遇事找法、解决问题用法、化解矛盾靠法的良好法治环境。坚持严格规范公正文明执法，推进服务型行政执法，决不允许以权代法、以权压法、逐利违法、徇私枉法。

严明纪律守规矩。“欲知平直，则必准绳；欲知方圆，则必规矩。”我们将切实把纪律和规矩挺在前面，严守党风廉政和改进作风的各项规定，持续落实中央“八项规定”精神及实施细则，驰而不息纠正“四风”，做到心有所畏、言有所戒、行有所止。严格落实党风廉政建设责任制，落实政府系统全面从严治党主体责任，加强政府系统廉政教育和廉政文化建设，努力打造忠诚、干净、担当的公务员队伍。

改进作风抓落实。“作风建设永远在路上，永远没有休止符。”我们将始终发扬“咬定青山不放松”的韧劲，讲政治、勇担当、马上就办、一抓到底，将“三·五”基层工作日、“六位一体”工作推进机制落到实处。加强政府智库建设，以科学决策引领科学发展。认真落实“基层减负年”要求，压缩会议、文件30%以上，集中清理各类评比达标表彰事项，优化改进各种督查检查、考核和调研活动，不干扰基层正常工作。关心基层干部的生活，为担当的干部担当，为负责的干部负责，形成人人尽责、务实重干的良好局面。

各位代表！商丘发展的前景十分美好，面临的发展任务十分繁重。让我们在市委的坚强领导下，高举习近平新时代中国特色社会主义思想伟大旗帜，不忘初心、砥砺前行，以坚如磐石的信心、只争朝夕的劲头、坚韧不拔的毅力，奋力谱写中原更加出彩商丘浓墨重彩的新篇章，以优异成绩庆祝中华人民共和国成立70周年！
